# The Risks of Downplaying Top-Down Control

**DOI:** 10.5334/joc.26

**Published:** 2018-05-14

**Authors:** Caitlin A. Sisk, Roger W. Remington, Yuhong V. Jiang

**Affiliations:** 1 Department of Psychology, University of Minnesota, US

**Keywords:** Attention, Reward processing, Visual search, Spatial cognition

## Abstract

Is top-down control necessarily scarce, slow, and hence unimportant in visual selection? Here we outline the risks of downplaying top-down control. Contrary to Theeuwes’ review, we suggest that not all sources of attention map onto a unitary attentional priority map. Goals and search habits may influence where and how people deploy attention, respectively. Because goals have modulatory effects on sensory processing, their impact on attention is broad and not always deliberate. In addition, when multiple sources influence attention, top-down control often dominates over less deliberate forms of attention. We agree with Theeuwes that selection history can drive attention independent of explicit goals. Nonetheless, top-down control remains a cornerstone of visual selection.

In the target article, Theeuwes (2018) builds on the framework of Awh, Belopolsky, and Theeuwes (2012), arguing that selection history constitutes a pathway for attentional control separate from the top-down/bottom-up dichotomy. Theeuwes proposes a new dichotomy between a fast, automatic form of control driven by stimulus salience and modulated by selection history, and a slow, volitional system driven by behavioral goals. Underlying this conception are three assertions: (1) all forms of attention act by modulating activity in a common spatial map; (2) goals play no role in modulating activity in the fast, automatic system; and (3) slow, volitional control is not only less frequent but also less important than fast, automatic control. We concur with Theeuwes on the importance of selection history in guiding attention. However, evidence provided here calls these assertions into question.

First, the assumption that attentional control is a product of activation within an integrated priority map obscures the nuanced mechanisms by which selection history drives attention. Holding the priority of each location on a spatial map equivalent, attentional biases can occur via procedural tuning. Recently, we showed that whereas top-down goals likely affect where people direct attention, perhaps in a manner akin to that described by the priority map, implicit location probability learning biases search by changing the preferred vector of attentional shift (Jiang, 2017). The idea that different sources of attention may not tap into a unified priority map is consistent with the observation that implicitly learned attentional biases resist generalization across different tasks. This lack of transfer is more in keeping with procedural habits than would be expected if all sources of attention project onto a common priority map.

Second, as Theeuwes discusses, the idea that behavioral goals cannot modulate attentional capture by salience directly contradicts contingent attentional capture (Folk, Remington, & Johnston, 1992) and search modes (Bacon & Egeth, 1994). Contingent capture presumes that behavioral goals establish a set that specifies which features lead to efficient attention allocation. Theeuwes interprets previous findings of contingent capture as reflecting inter-trial repetition priming. This argument, however, neglects studies that have shown patterns of attention capture determined by changes in search mode, with no change in stimulus sequence that would influence inter-trial priming (e.g., Becker, Folk, & Remington, 2013; Irons, Folk, & Remington, 2011; Wu & Remington, 2003). In addition, Theeuwes’ dichotomization of the fast and slow systems hinges on a narrow definition of top-down control – that it is necessarily volitional. In contrast, the contingent capture theory postulates that attentional control settings can occur without deliberate intent. This postulation is consistent with the larger literature on cognitive control. Extensive evidence suggests that dormant goals spontaneously intrude into ongoing activities, suggesting that goal states are not always under volitional control. On this broader definition of top-down control, even automatic processes are influenced by the observer’s goal state.

Finally, we must address the assumption that due to its scarcity, top-down control is less important in driving attention than fast, automatic influences. Even were it less frequent than experience- or salience-driven attention, the idea that top-down control is therefore less important does not directly follow. This logical jump fails to consider the relative magnitudes of the effects of types of control. In Cherry’s work on dichotic listening, for example, only 33% of participants attended to the ignored channel when their name was mentioned, whereas 80% did when told that new instructions may appear on the ignored channel (Pashler, 1998). Furthermore, in comparable paradigms, goal-driven attention yields an effect twice as large as statistical learning, which in turn trumps the magnitude of reward-driven spatial selection (Jiang, Sha, & Remington, 2015). In fact, the presence of goal-driven attention overshadows effects of selection history (Jiang, Swallow, & Rosenbaum, 2013). Thus, even if one accepts the claim that deliberate goals seldom control attention, it is important to recognize that when it is engaged, goal-driven attention has a predominating impact on behavior.

In summary, Theeuwes offers a new dichotomy of fast versus slow control of attention, grouping selection history and bottom-up attention into the category of fast, automatic control. We believe that compelling evidence supports the idea that selection history may influence attention quickly. However, the relegation of top-down control to a slow, volitional system incapable of modulating bottom-up effects and unimportant to daily life is at odds with existing data. Theeuwes provides a well-articulated framework that reduces ambiguity in terminology. Such clarity will steer attention research toward an understanding of how these diverse sources interact to optimize visual selection.

## Data Accessibility Statement

The authors have no data accessibility statement to declare.
